# Comparative study on the distribution of Pacinian corpuscles in the pancreas

**DOI:** 10.3389/fnana.2025.1593682

**Published:** 2025-08-13

**Authors:** Ting Yang, Ke Ren, Xiangquan Chen, Taku Toriumi, Rujia Li, Jun Li, Konosuke Tokita, Shuang-Qin Yi

**Affiliations:** ^1^Department of Frontier Health Sciences, Graduate School of Human Health Sciences, Tokyo Metropolitan University, Tokyo, Japan; ^2^Faculty of Physical Education, Qu Jing Normal University, Yunnan, China; ^3^Department of 1st Anatomy, Nippon Dental University School of Life Dentistry at Niigata, Niigata, Japan; ^4^Department of Hepatobiliary Surgery, Gansu Provincial Hospital, Lanzhou, China; ^5^State Key Laboratory of Oncogenes and Related Genes, Shanghai Cancer Institute, Ren Ji Hospital, School of Medicine, Shanghai Jiao Tong University, Shanghai, China; ^6^Department of Physical Therapy, Faculty of Health Sciences, Saitama Medical University, Saitama, Japan

**Keywords:** pancreas, Pacinian corpuscles, vibration-sensitive mechanoreceptor, pressure mechanoreceptor, cynictis penicillata

## Abstract

**Background:**

Pacinian corpuscles (PCs) are pressure- and vibration-sensitive mechanoreceptors found in hairless skin, external genitalia, joints, ligaments, lymph nodes, prostate, bladder, etc. While they are documented in the pancreas of cats, their presence in the normal pancreas remains speculative.

**Purpose:**

The present study therefore investigated the distribution of PCs in the normal human pancreas and compared the findings with those in several other animal species.

**Methods:**

The study subjects included 74 human cadaver specimens, 3 *Cynictis penicillata*, 2 *Saguinus mystaxs*, 1 *Felis domesticus*, and 10 *Suncus murinus*. Pancreatic tissues were prepared as paraffin sections for histological and immunohistochemical analyses of the main constituents of PCs (central axon, inner core, and outer core capsule).

**Results:**

PCs were found in the pancreas of five human cadavers (7%), as well as in one *C. penicillata*, one *S. mystax* and one *F. domesticus* but not in *S. murinus*. The PCs varied in size, with the largest in the human pancreas measuring up to 1,106 μm—far exceeding those in animal pancreata, but less numerous than those in animals. Morphologically, animal PCs were mainly typical oval shapes, whereas PCs in the human pancreas were mostly irregular in shape. In addition, we found that PCs in animals and human pancreata had similar structures, with consistent expression of protein gene product 9.5, in axonic profiles, and diffuse vimentin immunoreactivity in the inner core, outer core, and capsule.

**Conclusion:**

This study confirmed the presence of PCs in a small number of healthy humans and some animal pancreata. The number, distribution characteristics, and morphology of PCs in the pancreata of animals and humans are quite different; however, their structures and immunohistochemical profiles are similar. The presence of PCs in the normal human pancreas is also a mystery, and the physiological role of PCs in the human pancreas requires further clarification.

## 1 Introduction

The presence of Pacinian corpuscles (PCs) in the nerves of the fingers was first discovered in 1741 by Abraham Vater and later described histologically in detail by the Italian anatomist Filippo Pacini in 1830 before being designated according to him in 1835 (Cauna and Mannan, 1958). PCs are most often present in glabrous skin, notably on both surfaces of the hands and feet, they typically occur in large numbers and tightly grouped (Cauna and Mannan, 1958; Germann et al., 2021). They are also widely found in various tissues and organs throughout the human body, such as in connective tissues near tendons and joints (Halata et al., 1985; Zimny and Wink, 1991), interosseous membranes of the forearm and leg (Stecco et al., 2006), fascia (Stecco et al., 2007), urinary bladder (Landon and Wiseman, 2001), external genitalia (Martín-Alguacil et al., 2011), adventitia of large vessels (femoral artery and deep blood vessels in the upper arm) (Morishita et al., 2018; Onishi et al., 2021), prostate (Pai, 2017; de Souza and Athanazio, 2022), lymph nodes (Polski and Spreen, 2005; Aydin, 2006; Feito et al., 2017; Uguen, 2018; Gupta and Ojha, 2020) and thymus (Varga et al., 2020). Some authors have also described their unusual presence in the pancreas, particularly in cats (Krause, 1880; Kawahara, 1952; Iwanaga et al., 1982; Kumamoto et al., 1988; Liu et al., 1994; Al-Saffar and Al-Zuhairy, 2017). However, while histology textbook (Campbell and Verbeke, 2013) has also documented the presence of PCs in the human pancreas, just three such cases have been mentioned in two reports so far, all of which were from pancreatic cancer specimens (Standop J. et al., 2001; García-Suárez et al., 2010).

PCs have been reported to have various shapes, such as spherical, elliptical, oblong, cylindrical, and cucumber shapes, but the elliptical shape accounts for the majority of cases (Cauna and Mannan, 1958; Kumamoto et al., 1988). It has also been reported that the size, shape, and structure of PCs shift with age, showing considerable variations in shape and size, even in a single individual (Cauna and Mannan, 1958). Structurally, PCs with a central axon containing an unmyelinated nerve terminal and periaxonal cells form three distinct compartments: the inner core, outer core, and capsule (Bell et al., 1994; Ziolkowski et al., 2024). Nowadays, immunohistochemical analyses show that each PC component has its own unique expression. The central axons have been shown to be immunoreactive for panneuronal markers protein gene product (PGP) 9.5 (García-Suárez et al., 2010), neurofilaments protein (NFP) (Feito et al., 2017), neuronspecific enolase (NSE) (Standop J. et al., 2001; Feito et al., 2017), and synaptophysin (Varga et al., 2020). In addition, the inner bulb showed immunoreactivity for CD56 (Varga et al., 2020), S100 protein (S100P) (García-Suárez et al., 2010; Feito et al., 2017; Varga et al., 2020), cytochrome P450 (CYP) (Standop J. et al., 2001), glial fibrillary acidic protein (GFAP) (García-Suárez et al., 2010), and vimentin (García-Suárez et al., 2010). Furthermore, cytochrome P450 (CYP) (Standop J. et al., 2001), vimentin (García-Suárez et al., 2010), type IV collagen and glucose transporter 1 (Feito et al., 2017) were also observed in the outer core and capsule.

PCs are well known as rapidly adapting low-threshold mechanoreceptors and are responsible for the transduction of relatively high-frequency tactile perception of mechanical vibrations (Cauna and Mannan, 1958; Bell et al., 1994; Bouley et al., 2007). PCs are also involved in the regulation of blood pressure in blood vessels; around blood vessels, they sense changes in pulsating vibration due to flexion/extension of the joints (Morishita et al., 2018; Onishi et al., 2021). In the lymph nodes, the presence of capillaries in the outer core and capsule of the lymph nodes has led many researchers to hypothesize their role in vascular regulatory functions (Ceelen, 1912; Polski and Spreen, 2005; Feito et al., 2017; Gupta and Ojha, 2020). PCs may also be found in unusual anatomical locations and are considered anomalous, such as in the prostate or bladder, where their occurrence may be unexpected, leading to diagnostic confusion. They may also be confused clinically with tumor deposits (Dixon et al., 1992; Landon and Wiseman, 2001; Pai, 2017; Medlicott et al., 2019; Eccles and Hemmings, 2021). However, since they are also found in various parts of the body, their function remains the subject of considerable speculation, even today.

In our systematic histopathological studies of pancreatic lesions, such as pancreatic fibrosis and pancreatic fatty degeneration in cadaver human pancreata (Chen et al., 2023a; Li et al., 2024a), we unexpectedly observed PCs in serial sections of cadaver human pancreatic tissue. In the present study, we further explored the occurrence of PCs in the pancreas of all cadaver pancreatic specimens available in our laboratory and expanded to other animal species, including *Felis domesticus* (domesticated cats) and *Cynictis penicillata* from the Feliformia family of Carnivora, *Saguinus mystax* from the Cebidae family of Primates, and *Suncus murinus* from the Soricidae family of Eulipotyphla. In addition, we analyzed and compared the distribution, structure, and immunohistochemical characteristics of PCs in the pancreata of humans and animals.

## 2 Materials and methods

### 2.1 Cadaver pancreatic samples

Human pancreatic tissue specimens were obtained from cadavers [*n* = 74 (28 males, 46 females); mean age at the time of death, 87.7 (range, 58–106) years old] used for anatomy research and education at the School of Life Dentistry, Nippon Dental University in Tokyo, and Niigata from 2014 to 2023 (Supplementary Table 1). All cadavers were fixed and preserved in formalin for approximately 6 months. Intact whole pancreatic tissue was removed from the cadavers, and all selected specimens were confirmed to be well fixed with the naked eye. Cadavers with abdominal injuries or severe abdominal pathology (especially partial loss of the pancreas, deformity, pancreas-related causes of death, and adhesions between the pancreas and surrounding organs), were excluded from this study.

All study procedures were approved by the Study Security and Ethics Committees of Tokyo Metropolitan University (No.18051), Nippon Dental University in Tokyo (NDU-T-2016-29), and Niigata (No. 23823). This study was performed according to the institutional guidelines.

### 2.2 *S. murinus*

*S. murinus* (*n* = 10, age range 4–6 months) housed in a closed breeding colony (JIc: KAT-c) in our laboratory (Functional Morphology Laboratory, Department of Frontier Health Sciences, Tokyo Metropolitan University, Tokyo, Japan) were used in this study. Adult *S. murinus* were kept in an experimental animal facility at room temperature (RT) of 25–28°C, under a 12 h light and dark cycle, with ad libitum access to trout chow and water (Li et al., 2024b).

All animal experiments were approved by the Institutional Animal Care and Use Committee of Tokyo Metropolitan University (Permit Numbers: A4-26, A5-17, and A6-15). All experimental procedures were performed in accordance with the National Research Council Guide for the Care and Use of Laboratory Animals.

### 2.3 *F. domesticus*, *C. penicillata*, *S. mystax*

*F. domesticus* (*n* = 1), *C. penicillata* (*n* = 3), and *S. mystax* (*n* = 2) were obtained from the specimen bank of our laboratory. The researchers in this laboratory collected animals that died of natural causes from the city’s zoos and preserved them in formalin without decay.

### 2.4 Preparation of tissue specimens

Human pancreatic tissue samples were processed as previously described (Li et al., 2024b). To obtain well-preserved sections, pancreatic specimens obtained from cadavers were divided into three anatomical regions: the head (including the uncinate process), body, and tail. The specimens were cut into 5-mm-thick tissue blocks, prepared in 20–27 blocks per pancreas, which collectively covered all three regions. And then washed thoroughly for 4–5 h under running tap water and routinely embedded in paraffin, with a 5-μm-thick section cut from each and placed on a gelatin-coated glass slide. For histological analysis, five or more sections were prepared and examined per block. Each specimen underwent hematoxylin and eosin (HE) staining and, when necessary, immunohistochemical staining were performed, followed by a microscopic examination.

*S. murinus* specimens were first euthanized under deep anesthesia with isoflurane inhalation, the thoracic cavity was opened, and a catheter was inserted into the left cardiac ventricle while the right atrium was simultaneously opened. Subsequently, perfusion with phosphate-buffered saline (PBS; 0.01 M, pH 7.4) and 4% paraformaldehyde (PFA) were performed. *S. murinus* was then immersed and fixed in 4% PFA at 4 °C overnight. The next day, pancreatic tissues were dissected and divided into the cephalic (close to the duodenum) and caudal (close to the spleen) sides. After rinsed thoroughly under running tap water for 4–5 h, tissues were dehydrated and paraffin-embedded. Five microgram sections were mounted on gelatin-coated glass slides, and at least five sections per block were subjected to histological or immunohistological analyses, as with the human samples.

*F. domesticus*, *C. penicillata*, and *S. mystax* specimens collected in our laboratory were fixed well. The entire pancreas specimen was segmented into the cephalic and caudal parts and paraffin-embedded tissue sections were prepared, and at least five sections per block were subjected to histological or immunohistochemical analyses as described above for *S. murinus*.

### 2.5 Histological and immunohistochemical analyses

After HE staining of pancreatic specimens from cadavers and animals, we systematically analyzed at least five sections per specimen to ensure representative sampling. Quantitative assessments included: PCs enumeration performed at 40 × magnification, with calculation of mean PCs density per section. For morphometric analysis of PCs dimensions, given the characteristic onion-like lamellar architecture of PCs and their considerable cross-sectional variability, the maximum diameter of the largest PC per specimen (rather than mean values or size ranges) based on multi-sectional observations. All dimensional measurements were performed at 200 × magnification using calibrated ocular micrometers, documenting the largest structurally intact PC (defined by unequivocal visualization of the concentric inner core, outer core, and complete capsular layers) in each specimen. In addition, we studied these sensory corpuscles using immunohistochemistry analyses specific for each of the main corpuscular constituents, i.e., the central axon, inner core, and outer core capsule.

Immunohistochemical analyses were performed as previously described (Li et al., 2024a), and tissue sections were deparaffinized with xylene, rehydrated in a graded ethanol series, washed under running tap water, and rinsed in 0.01 M TBST (Tris-buffered saline containing 0.1% Tween 20; pH 7.6). Antigen retrieval was performed by pretreatment at high temperature and pH 6.0 in citrate buffer. Endogenous peroxidase activity was inhibited by incubating with methanol containing 0.3% (v/v) hydrogen peroxide for 20 min. To block nonspecific binding, the sections were treated with 5% bovine serum albumin containing 3% goat serum for 1 h at room temperature (RT), and the sections were incubated overnight in a humidified chamber at 4 °C with the primary antibody. Antibodies were diluted in TBS (pH 7.6) containing 5% bovine serum albumin (BSA). The sections were then rinsed in the same buffer and incubated with the secondary antibody for 1 h at RT. Finally, the sections were washed, and the immunoreaction was visualized using 3–3′-diaminobenzidine as a chromogen.

The primary antibodies used in the experiments were polyclonal antibodies, anti-PGP 9.5/UCHL1 (1:50, rabbit, 14730-1-AP; Proteintech, Tokyo, Japan) as a central axon marker, and anti-Vimentin (1:1,000, rabbit, 10366-1-AP; Proteintech) as a maker for the inner and outer core capsules, all of which were diluted in 0.01 M TBS (pH 7.6) containing 5% BSA. The secondary antibody was anti-rabbit IgG-HRP (H+L chain) (No. 458; MBL, Tokyo, Japan) diluted at 1:100 in TBS (pH 7.6) containing 5% BSA.

For control purposes, representative sections were processed in the same way as described previously, using non-immune rabbit sera instead of the primary antibodies or omitting the primary antibodies during incubation.

### 2.6 Masson’s trichrome analyses

Masson’s trichrome staining was performed as previously described in a previous study (Chen et al., 2023a). In brief, we performed the following: (1) deparaffinization and rehydration (xylene for 10 min, 3 times; 100% ethanol for 5 min, 3 times; 95% ethanol for 5 min; 90% ethanol for 5 min; 80% ethanol for 5 min; 70% ethanol for 5 min; tap water for 10 min), (2) hematoxylin staining (hematoxylin applied for 8 min; rinsed with distilled water; immersed for 25 s in 70% alcohol and 1% hydrochloric acid to de-stain; rinsed with distilled water), (3) Biebrich Scarlet taining (Biebrich Scarlet applied to the slid for 2 min; rinsed with distilled water), (4) phosphomolybdic-phosphotungstic acid differentiating (2 h), and (5) aniline blue staining and dehydration (aniline blue applied for 35 min; rinsed with distilled water; immersed for 30 s in 1% acetic acid, rinsed with distilled water; immersed in 95% ethanol for 45 s; 100% ethanol for 5 min, 3 times; and xylene for 5 min, 3 times).

### 2.7 Statistical analyses

Statistical analyses were performed using the IBM SPSS Statistics software program (version 29.0; IBM, Armonk, New York, United States). Comparisons of general characteristics were using the *t*-test for age and Fisher’s exact test for gender. A *p*-value < 0.05 was considered statistically significant.

## 3 Results

The number, distribution characteristics, and morphology of PCs in the pancreata of humans and other animal species were summarized in Table 1.

**TABLE 1:** The number, distribution characteristics and morphology of PCs in the pancreata of human and other animal species.

Species	Number* (mean ± SD)	Size** (μm)	Distribution characteristics	Histological features		
				**Shape**	**Number of IC**	**Number of OC**
Humans (cadaver)	1–3 (1.6 ± 0.9)	1,106	Pancreatic subserosa, adjacent to the pancreatic lobes	Long spindle, irregular polygon, triangular pyramid	1∼2	1∼2
*C. penicillate*	5–7 (5.8 ± 0.8)	603.3	Intralobular or interlobular areas, near the pancreatic duct or artery	Oval	1 (most)∼2	1 (most)∼2
*S. mystax*	10–15 (12.4 ± 2.1)	659.9	Intralobular or interlobular areas not adjacent to the pancreatic duct or artery	Oval	1∼3	1∼3
*F. domesticus*	31–38 (34.0 ± 3.3)	695.8	Intralobular or interlobular areas, near the pancreatic duct or artery	Irregular oval	1∼2	1∼2
*S. murinus*	–					

IC, inter core; OC, outer core.

*Number of Pacinian corpuscles observed for species.

**Size (maximum diameter) of the largest Pacinian corpuscles identified in each species.

### 3.1 PCs in pancreatic tissue of cadavers

**FIGURE 1:**
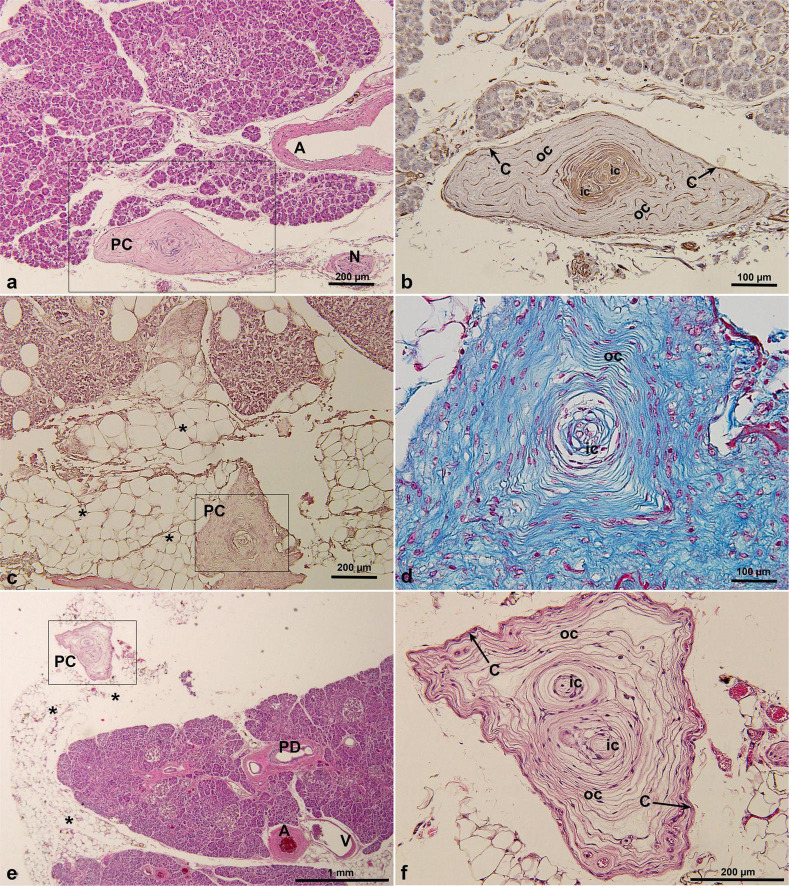
PCs in cadaveric pancreatic tissue by HE staining **(a,c,e,f)**, Masson’s trichrome staining **(d)** and immunohistochemical staining of vimentin antibody **(b)**. PCs were located under the pancreatic serosa, adjacent to the pancreatic lobe, and were irregular polygonal or triangular pyramidal in shape. *, indicating pancreatic fatty degeneration in **(c)** and fat tissue surrounding pancreas in **(e)**. A, artery; C, capsular cells; IC, inner core; N, nerve; OC, outer core; PC, Pacinian corpuscle; PD, pancreatic duct; V, vein.

PCs were identified in 5 of 74 examined cadaver pancreatic specimens (prevalence: 7%), and their corresponding ages, genders and causes of death were as shown in Table 2 and Supplementary Table 1. Among the five human pancreatic specimens containing PCs, we observed a mean of 1.6 ± 0.9 PCs per case (range: 1–3). The maximum diameter of the largest PC identified was 1,106 μm. Some of their locations and structures varied, in one case, the PC was located in the subserosa of the pancreas and closely adjacent to the pancreatic tissue, which was different from the general oval shape but took on a long spindle shape (Figure 1a). PCs in the human pancreas had two distinct cores, and the immunohistochemical profile of the PCs was analyzed, with the inner core, outer core, and capsular cells displaying immunoreactivity for vimentin (Figure 1b). In another case, the PC was located in the adipose tissue adjacent to pancreatic fatty degeneration and also presented a special shape, with a triangular cross section (Figure 1c), while retaining the same structure of the inner core, outer core, and capsule. Masson’s trichrome staining revealed that the lamellar composition of the inner core, outer core, and capsular cells appeared the same blue as in the collagen fibers, while the fibroblasts appeared red (Figure 1d). In another case, the PC was located slightly distal to the pancreatic parenchyma within the subserosal adipose tissue of the pancreas, which also had a very different shape and structure (Figure 1e). In the enlarged image, the PC showed an irregular polygonal shape or triangular-cone shape; structurally, it had two different inner and outer cores and was wrapped in the same capsule (Figure 1f).

**TABLE 2:** General information of human pancreatic PC cases.

No list	No of the cadavers	Sex*	Age*	Cause of death
1	1,489	M	93	Aspiration pneumonia
2	1,651	F	91	Senility deaths
3	N993	M	86	Pneumonia
4	S1002	M	85	Cerebral infarction, chronic atrial fibrillation, chronic heart failure
5	S1008	F	98	Senility deaths

*There was no significant difference in age and gender distribution between these five Pacinian corpuscle cases and the other 69 cases.

In addition, demographic analysis revealed no significant association between PC occurrence and age or gender. The five PC cases had a mean age of 90.6 ± 2.4 years, showing no significant difference compared to the remaining 69 cases in either age (*P* = 0.502) or sex distribution (*P* = 0.36) (Table 2).

### 3.2 PCs in pancreatic tissue of *C. penicillata*

**FIGURE 2:**
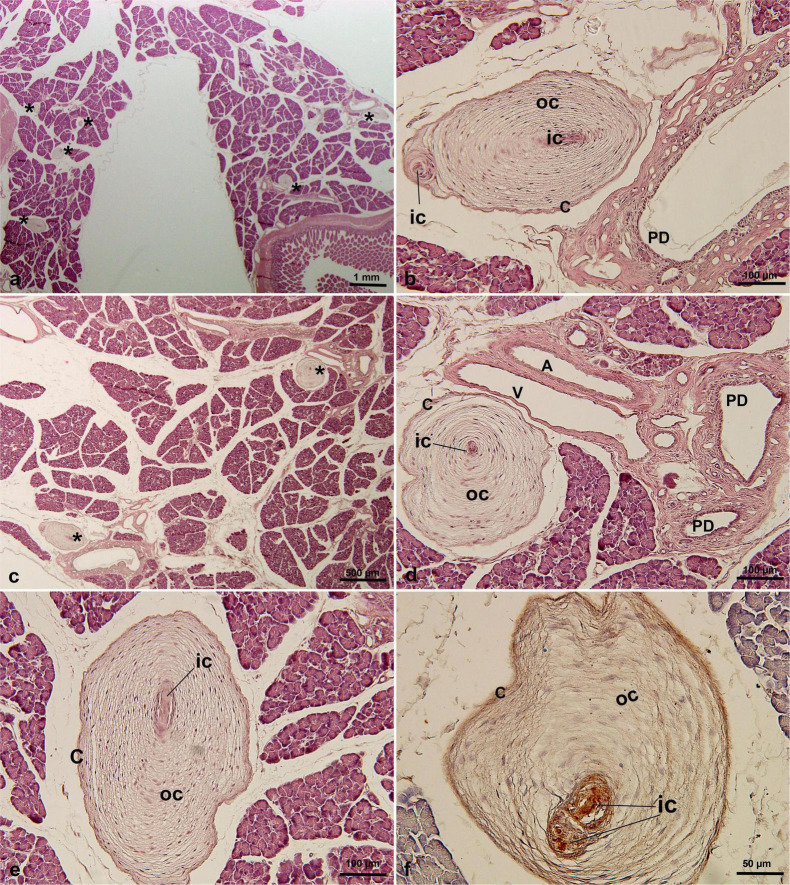
PCs in *C. penicillata* pancreatic tissue by HE staining **(a–e)** and immunohistochemical staining of vimentin antibody **(f)**. PCs were oval in shape and multiple PCs existed in the same slice. PCs were distributed in the intralobular or interlobular areas, usually adjacent to the pancreatic ducts or blood vessels. * in **(a,c)**, indicating Pacinian corpuscles. A, artery; C, capsular cells; IC, inner core; OC, outer core; PD, pancreatic duct; V, vein.

PCs were found in only one of three cases of pancreatic tissue from *C. penicillata*. Based on the analysis of multiple histological sections, an average of 5–7 PCs per section was observed (mean 5.8 ± 0.8). The maximum diameter of the largest PC detected was 603μm (Table 1). These PCs were distributed throughout the pancreatic parenchyma (Figures 2a,c), with most PCs were located between the pancreatic lobules (Figures 2e,f), and some PCs were located close to the pancreatic ducts and/or blood vessels (Figures 2b,d). In addition, they were mainly oval in shape (Figures 2a,c). Typical concentric structures were composed of a single inner core, outer core, and capsule (Figures 2d,e), but there were also structures with multiple inner and outer cores (Figures 2b,f). An immunohistochemical analysis showed that the inner core, outer core, and capsule were positive for vimentin (Figure 2f).

### 3.3 PCs in pancreatic tissue of *S. mystax*

**FIGURE 3:**
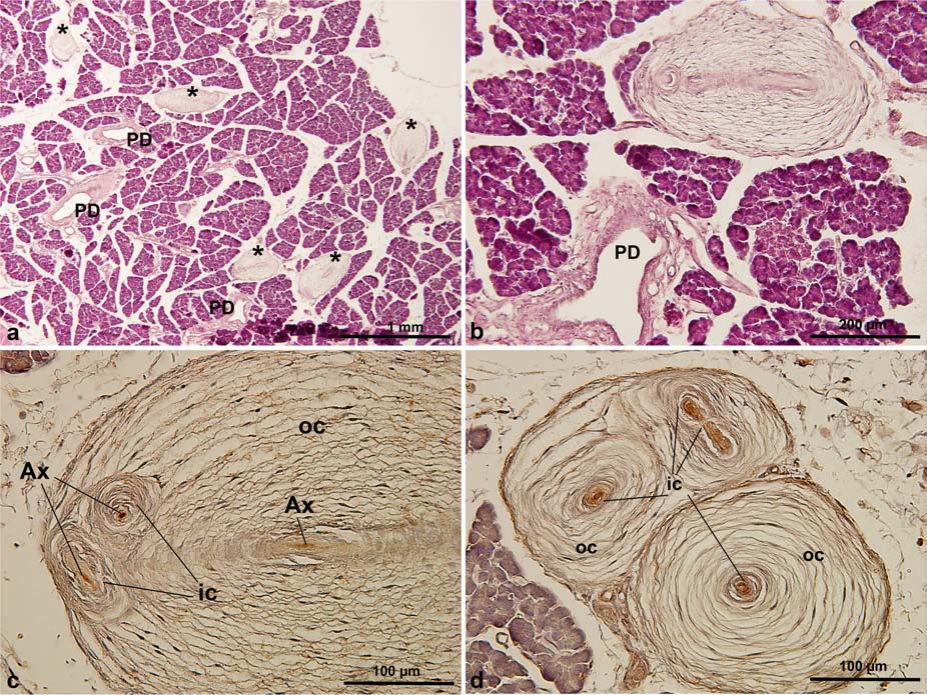
PCs in *S. mystax* pancreatic tissue by HE staining **(a,b)** and immunohistochemical staining of PGP 9.5 **(c)** and vimentin **(d)** antibodies. PCs were distributed within or between lobules, were generally not adjacent to the pancreatic ducts or blood vessels, were oval in shape, and were distributed in large numbers. * in **(a)**, indicating Pacinian corpuscles. AX, axon; IC, inner core; OC, outer core; PD, pancreatic duct.

Histological analysis revealed that PCs were present in only one of the two *S. mystax* pancreatic specimens, with a density of 12.4 ± 2.1 PCs per section (range: 10–15), with a maximum observed PC diameter of 660 μm (Table 1). Numerous PCs were scattered within the lobules of the pancreas (Figure 3a) and these PCs were not closely associated with the blood vessels or pancreatic ducts (Figure 3b). They were also mainly oval in shape, with multiple inner and outer cores in a single PC (Figures 3b–d). The central axon displayed immunoreactivity for PGP 9.5 (Figure 3c), and similar to PCs in humans and *C. penicillata*, the inner core, outer core, and capsule were all immunoreactive for vimentin (Figure 3d).

### 3.4 PCs in pancreatic tissue of *F. domesticus*

**FIGURE 4:**
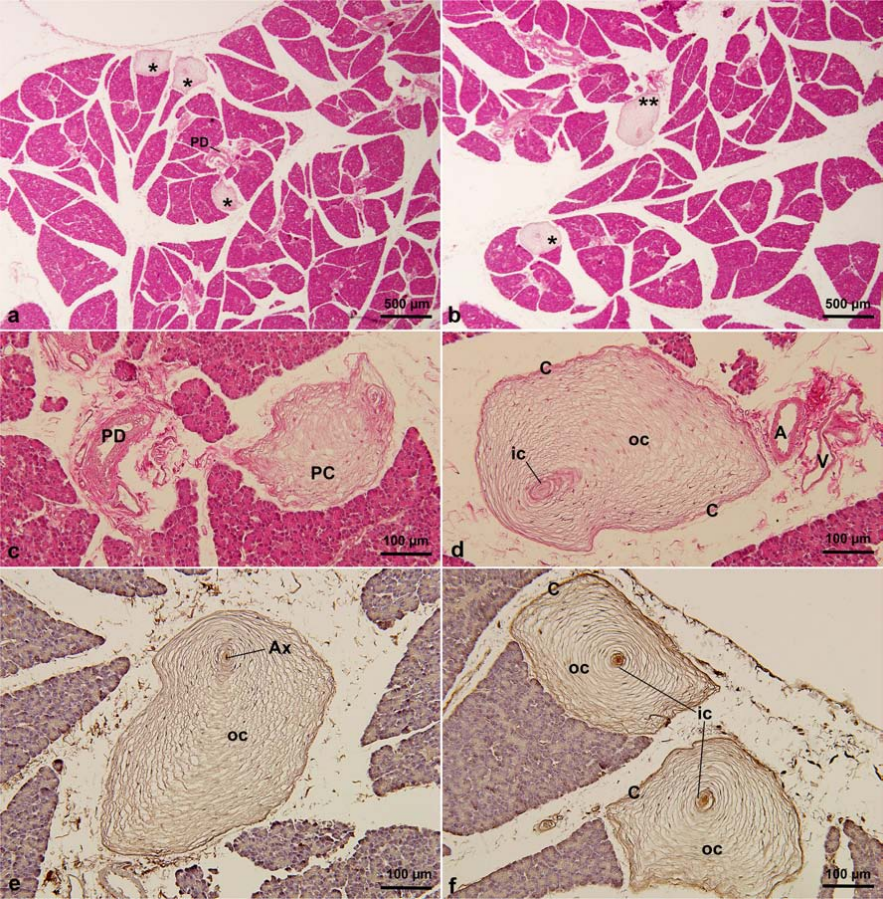
PCs in *F. domesticus* pancreatic tissue by HE staining **(a–d)** and immunohistochemical staining of PGP 9.5 **(e)** and vimentin **(f)** antibodies. PCs were distributed among the pancreatic lobules, and some were adjacent to the pancreatic ducts or blood vessels. PCs were irregularly oval in shape and numerous. * in **(a,b)**, indicating Pacinian corpuscles. A, artery; AX, axon; C, capsular cells; IC, inner core; OC, outer core; PC, Pacinian corpuscle; PD, pancreatic duct; V, vein.

A relatively high number of PCs was observed in the *F. domesticus* pancreatic tissue, with a density of 34.0 ± 3.3 cysts per section (range: 31–38) (Table 1). And they were scattered in the intralobular or interlobular regions of the pancreas (Figures 4a,b). The largest identified PC measured 696 μm in maximum diameter (Table 1). Some PCs were also distributed near the pancreatic ducts (Figure 4c) or close to the blood vessels (Figure 4d). Although the shape was not exactly a typical oval (irregular oval), most had four typical structures: a central axon, inner core, outer core, and capsule. Furthermore, the immunohistochemical expression profiles of the analyzed PCs were identical, with the central axon showing positivity for PGP 9.5 (Figure 4e), whereas the remaining three structures (inner core, outer core, and capsule) were positive for vimentin (Figure 4f).

### 3.5 PCs in pancreatic tissue of *S. murinus*

In the present study, we also performed a histopathological analysis of the pancreatic tissue from 10 *S. murinus* specimens, but no PCs were observed in these tissues (Supplementary Figure 1).

## 4 Discussion

In the present study, we performed histopathological analyses of the local distribution of PCs in pancreatic tissues from 74 cadavers and 3 *C. penicillata*, 2 *S. mystaxs*, 1 *F. domesticus*, and 10 *S. murinus* specimens. As shown in Supplementary Table 1 and Table 1, PCs were present in the pancreatic tissue of five cadavers and one *C. penicillata*, one *S. mystax*, and one *F. domesticus* specimen but not in any *S. murinus* specimens. Observations revealed that multiple PCs were typically clustered together, though a single PCs was occasionally observed. In addition, the difference in the size of the PCs was very marked, with some being twice as large as others. In terms of quantity, it was clear that PCs were more densely packed in the pancreatic tissue of *C. penicillata*, *S. mystax*, and *F. domesticus* than in the human pancreas. Furthermore, the location and shape of PCs in the human pancreas and in different animal species were also different. Structurally, most PCs of these species were clearly identifiable, although there were similarities in the development of the inner core, number of lamellae forming the outer core capsule, and density and packaging of the capsular lamellae. These morphological differences may imply subtle differences in the function of PCs in different species.

In the present study, histological analyses of 74 normal human pancreatic specimens revealed the presence of PCs in five cases (7%), primarily located in the interlobular connective tissue or subserosal regions. To date, PCs have been only sporadically documented in human pancreatic tissue. In the early 20th century, Ceelen (1912) first reported the presence of PCs in the pancreas, observed in 89% of human postmortem examinations. Although Wikipedia, subsequent histological textbook (Campbell and Verbeke, 2013) and references have described the presence of PCs in human pancreatic tissue, contemporary studies remain scarce. Only few reports have demonstrated the presence of PCs in the pancreas of three patients with pancreatic cancer. Standop J. et al. (2001) PCs conducted a comprehensive histological analysis of 40 pancreatic specimens (20 normal samples from organ donors and 20 tumor resection specimens from pancreatic cancer patients) and only identified a single PC in the connective tissue of one pancreatic cancer resection specimen (detection rate: 2.5%). Similarly, García-Suárez et al. (2010) reported PCs within the connective tissue of two pancreatic cancer resection specimens. More recently, a study by Proshchina et al. (2018) reported that the detection of PCs in 1 pancreatic section of a newborn diagnosed with diabetic fetal disease among more than 1,000 slices from 42 fetuses and newborns as well as 65 adults. These findings suggest that PCs may indeed be rare anatomical structures in the human pancreas. However, whether their occurrence is incidental or bears significant functional relevance remains to be elucidated through further investigation.

In addition, since the human pancreas specimens in this study were from elderly people who died of natural causes, their bodies were donated to the Department of Stomatology of the Faculty of Medicine for anatomical practice after death. Therefore, it is difficult for us to conclude the association between age and PCs from these five cases. It has been reported that PCs in diabetic monkeys have significant damage, including disintegration of the inner layer, irregular spacing, enlarged interlaminar spaces, rounded nuclei, and thickened outer layers (Paré et al., 2007). Unfortunately, the information we obtained about these specimens was limited (only the cause of death was known), and we were unable to conclude whether the appearance of PCs was related to their medical records during their lifetime.

In a series of histopathological studies on PCs in the pancreas of cats, it was reported that PCs are widely distributed in the pancreatic tissue of cats, mainly existing alone in the interlobular tissue, secondarily connected with the pancreatic ducts and blood vessels. Some exist alone, but others exist in groups of two to four. Most are oval and elliptical shape, with the longest diameters ranging from 0.14 to 1.32 mm (Kawahara, 1952; Iwanaga et al., 1982; Kumamoto et al., 1988; Liu et al., 1994). Our *F. domesticus* study findings were almost consistent with the previously reported results, and a greater number of PCs was found in *F. domesticus* pancreatic tissue than in humans. Furthermore, we also discovered a large number of PCs in the pancreas of animals other than cats for the first time, namely *C. penicillata* and *S. mystaxs*. However, no PCs were found in the pancreas of another 2 *C. penicillata*. Because *C. penicillata*, like cats, belong to the Feliformia family of the order Carnivora, the fact that PCs were present in the pancreas of *C. penicillata* is not difficult to believe. However, while *S. mystaxs* belongs to the Cebidae family of Primates, but still we did not detect any PCs in the pancreas of *Macaca fuscata*, members of the same family (data not shown). We also did not detect any PCs in the pancreas of *S. murinus* from the Soricidae family of the order Eulipotyphla. One study reported that PCs were found in the pancreas of four of five cats studied and suggested that cats with reduced PC numbers in the pancreas had large numbers of PCs in the mesentery and plantar pillows (Miclaus et al., 2011). It is possible that PCs are simply not present in the pancreata of all animals. The precise role of PCs in the pancreas requires a further evaluation.

A normal PC is an elliptical column or a spherical structure composed of 20–60 concentric layers, with the length of the corpuscle ranging from about 0.5 to 1.5 mm and an average length and width of approximately 1 and 0.7 mm, respectively (Cauna and Mannan, 1958; Bell et al., 1994). In cat pancreas, the longest diameter of PCs reportedly ranges from 0.14 to 1.32 mm (Kumamoto et al., 1988). In the present study, we found that the PCs in the animal pancreata were mostly elliptical, whereas those in the human pancreata were more diverse, consisting mostly of irregular polygonal shapes. The size of the PCs also varies, with some being twice as large as others within the same pancreatic tissue, and among species, the PCs in the human pancreas tend to be slightly larger than those in animal species. Previous studies have shown that PCs in the digital and mesentery show considerable variation in shape and size, even within a single individual, with a general oval but sometimes slightly flattened or irregular shape (Cauna and Mannan, 1958). Similarly, most PCs in the pancreas of cats are elliptical, with some showing special shapes, such as spherical, loofah-shaped, cylindrical, and cucumber shapes (Kumamoto et al., 1988). This is consistent with the diversity of PCs observed in pancreatic tissue. Regarding the size of PCs, the average length of a corpuscle at birth ranges from 500 to 700 μm, and the size increases gradually throughout life, reaching 3–4 mm in length. However, in persons over 70 years old, corpuscles show regressive changes, becoming smaller and irregular digitally (Cauna and Mannan, 1958). In our study, we found that the PCs in human pancreatic tissues were mostly irregular polygonal or triangular cone shape. Obviously, the PCs in the pancreas are different from those that receive pressure through circular or oval surface tension. The irregular or triangular cone morphology is not good at receiving pressure and generating surface tension, which then transmits pressure information. Therefore, we speculate that the functions of PCs in the pancreas might be different from those of other species, or the way they receive information may be different.

Some PCs in the pancreas of humans and other animal species in our study showed only one axon, outer core, and outer fibrous capsule, presenting a typical “onion bulb” appearance. As described in previous studies, PCs in other body localizations are typically characterized by three well-differentiated components: (a) a central axon, (b) an inner core formed by differentiated Schwann-like cells, and (c) a more or less developed multilayered outer core and capsule formed by differentiated perineurial cells (Cobo et al., 2021; Onishi et al., 2021). We also found that some of these PCs had multiple axonic and inner core profiles, and each axon was enclosed in a proper inner core. Some studies have demonstrated that most PCs in human or animal pancreas have numerous axonic and inner core profiles compared to PCs in the skin, and some axonic profiles each enclosed by their own inner core have been confirmed (Kawahara, 1952; García-Suárez et al., 2010). Whether true polyinnervation exists in these corpuscles or whether they represent the branches of a single main axon remains to be elucidated. Furthermore, our study showed that the immunohistochemical profiles of PCs in human and animal pancreata were identical, with axonic profiles invariably expressing PGP 9.5 and diffuse immunoreactivity for vimentin shared by the inner core, outer core, and capsule. These results are consistent with the immunohistochemical characteristics of PCs reported in other locations in the body (García-Suárez et al., 2010).

It is universally known that cutaneous PCs, as rapidly adapting low-threshold mechanoreceptors, are responsible for high-frequency (20–1,000 Hz) vibrations and have high-pass filtering and mechanical signal amplification functions. It is the main receptor of vibration tactility and is closely related to fine touch sensation, which is the ability to perceive and localize an object’s shape, texture, and size (Johnson, 2001; Zimmerman et al., 2014; Chen et al., 2023b). Therefore, many mammals can sense vibrations and their locations, such as one possible anatomical mechanism used by elephants to detect seismic waves (Bouley et al., 2007). Some studies have also shown that those located deep in the body can feel internal vibration stimulation, such as heartbeat, respiration, and blood flow (Sugai et al., 2021; Chen et al., 2023c). PCs have been found around blood vessels in various locations of the body, such as the lymph nodes, upper arm’s deep arteries, femoral artery, and vein (García-Suárez et al., 2010; Feito et al., 2017; Morishita et al., 2018; Onishi et al., 2021), and some scholars have viewed the function of these PCs as being connected with vascular tone and regulation of blood pressure (Morishita et al., 2018). One study reported that PCs accumulated near the elbow around the deep blood vessels that bend at elbow flexion, indicating their role in the detection of blood flow alterations at flexion and extension of the elbow (Onishi et al., 2021). PCs in the mesentery have been reported to signal the degree of distension of the mesenteric vessels or weight of the viscera (Bell et al., 1994). PCs have also been reported in lymph nodes, and were considered ectopic, to be an abnormal cicatrization process, possibly in response to trauma or other etiologies causing local inflammation (Polski and Spreen, 2005; Uguen, 2018). PCs have rarely been reported to be present in the bladder and prostate, where they are thought to be unique aberrations, and their function remains speculative (Dixon et al., 1992; Landon and Wiseman, 2001; Pai, 2017; Medlicott et al., 2019).

The present study analyzed the PCs in the pancreas of humans and animals and found that the distribution characteristics differed between the two, with humans being located in the interlobules (pancreatic interstitium) or subserosa of the pancreas. PCs in *C. penicillata* and *F. domesticus* pancreata are similar, as described by Kumamoto et al. (1988) and Al-Saffar and Al-Zuhairy (2017). The PCs were distributed throughout the pancreas, with some observed in the interlobular connective tissue, some of which were in contact with the adventitia of the pancreatic inter-lobular duct and others closely related to blood vessels. Kumamoto et al. (1988) speculated that, in the *F. domesticus* pancreas, in addition to functioning as external receptors, PCs close to the pancreatic duct and blood vessels may act as receptors inside the pancreas, sensing changes in the duct wall caused by changes in the pancreatic juice and blood flow. In the human pancreas, it has been postulated that recording the vibration and lymphatic flow, as well as the neuronal regulation of exocrine and endocrine secretion, detects peritoneal pressure, distension, and abdominal pain and regulates the function of the pancreas by regulating blood flow (Standop J. et al., 2001; García-Suárez et al., 2010). Although PCs are essentially native to nervous end-organs, their presence in the pancreas is mysterious. Proshchina et al. (2018) concluded that they may not play a significant role in the sensory innervation of the pancreas.

Although our findings suggest structural similarities in pancreatic PCs across species, the limited number of animal specimens precluded meaningful statistical analysis or definitive interspecies comparisons. Therefore, these observations should be interpreted as preliminary and descriptive, and further studies with larger sample sizes are required to validate these trends.

In conclusion, in the present study, we reported the presence of PCs in normal pancreas and found that although the number, distribution, and morphology of PCs differed between the pancreas of humans and other animal species, their structures were similar. However, the presence of PCs in the normal human pancreas still needs to be confirmed, and the functions of intrapancreatic and peripancreatic PCs and whether or not PCs are associated with the occurrence of pancreatic cancer remain unclear. Further investigation is needed to elucidate the physiological role of PCs in the human pancreas.

## Data Availability

The raw data supporting the conclusions of this article will be made available by the authors, without undue reservation.

## Supplementary material

Supplementary Figure 1 HE staining of the pancreas of *S. murinus*. No PCs were observed in the pancreas of *S. murinus*. La, islets of Langerhans; *, small islets of Langerhans; PD, pancreatic duct; A, artery; V, vein.

## References

[B1] Al-Saffar F. J. Al-Zuhairy M. F. (2017). Postnatal developmental Histomorphological and histochemical study of the pancreas in the domestic cat (Felis Catus). *Int. J. Advanced Res.* 5 55–71. 10.21474/IJAR01/3115

[B2] Aydin O. (2006). Pacinian corpuscle in a lymph node. *Neuropathology* 26 379–381. 10.1111/j.1440-1789.2006.00728.x 16961077

[B3] Bell J. Bolanowski S. Holmes M. H. (1994). The structure and function of Pacinian corpuscles: A review. *Prog. Neurobiol.* 42 79–128. 10.1016/0301-0082(94)90022-1 7480788

[B4] Bouley D. M. Alarcón C. Hildebrandt T. O’connell-Rodwel C. (2007). The distribution, density and three-dimensional histomorphology of Pacinian corpuscles in the foot of the Asian elephant (*Elephas maximus*) and their potential role in seismic communication. *J. Anat.* 211 428–435. 10.1111/j.1469-7580.2007.00792.x 17711421 PMC2375831

[B5] Campbell F. Verbeke C. S. (2013). *Pathology of the Pancreas: A Practical Approach.* London: Springer, 17. 10.1007/978-1-4471-2449-8

[B6] Cauna N. Mannan G. (1958). The structure of human digital Pacinian corpuscles (*Corpus culalamellosa*) and its functional significance. *J. Anat.* 92 1–20. 13513492 PMC1244958

[B7] Ceelen W. (1912). Veber das Vorkommen von vater-painischen koerperchen am menschlichen pankreas. *Virchow Arch.* 208 460–472. 10.1007/BF01991215

[B8] Chen J. Yang T. Nakagawa S. Li R. J. Zhang M. S. Yi S. Q. (2023a). Retrospective histopathological study of pancreatic fibrosis in cadaver samples. *J. Gastro. Hepato. V* 9 1–7. 10.2139/ssrn.4288092

[B9] Chen S. Li K. Qiao X. Q. Ru W. M. Lin X. (2023b). Tactile perception of fractal surfaces: An EEG-fNIRS study. *Tribol. Int.* 180:108266. 10.1016/j.triboint.2023.018266

[B10] Chen S. Yang Z. H. Huang Q. Li K. Ge S. R. (2023c). Vibrotactile sensation: A systematic review of the artificial Pacinian corpuscle. *J. Bionic. Eng.* 20 1401–1416. 10.1007/s42235-023-00348-8

[B11] Cobo R. García-Piqueras J. Cobo J. Vega J. A. (2021). The human cutaneous sensory corpuscles: An update. *J. Clin. Med.* 10:227. 10.3390/jcm10020227 33435193 PMC7827880

[B12] de Souza M. F. Athanazio D. A. (2022). Intraprostatic Pacinian corpuscle does exist! *Pathology* 54 479–480. 10.1016/j.pathol.2021.06.126 34565604

[B13] Dixon J. S. Gosling J. A. Canning D. A. Gearhart J. P. (1992). An immunohistochemical study of human postnatal paraganglia associated with the urinary bladder. *J. Anat.* 181 431–436. 1304581 PMC1259696

[B14] Eccles A. Hemmings C. (2021). Intra-abdominal Pacinian corpuscle mimicking a peritoneal tumour deposit. *Pathology* 53:939. 10.1016/j.pathol.2021.01.012 33941381

[B15] Feito J. Cobo J. L. Santos-Briz A. Vega J. A. (2017). Pacinian corpuscles in human lymph nodes. *Anat. Rec.* 300 2233–2238. 10.1002/ar.23679 28806498

[B16] García-Suárez O. Calavia M. G. Pérez-Moltó F. J. Alvarez-Abad C. Pérez-Piñera P. Cobo J. M. (2010). Immunohistochemical profile of human pancreatic pacinian corpuscles. *Pancreas* 39 403–410. 10.1097/MPA.0b013e3181bc0372 19910838

[B17] Germann C. Sutter R. Nanz D. (2021). Novel observations of Pacinian corpuscle distribution in the hands and feet based on high—Resolution 7-T MRI in healthy volunteers. *Skeletal. Radiol.* 50 1249–1255. 10.1007/s00256-020-03667-7 33156397 PMC8035111

[B18] Gupta C. Ojha S. (2020). Pacinian corpuscle in human lymph node: A report and the literature review. *J. Hematopathol.* 13 201–202. 10.1007/s12308-020-00396-7

[B19] Halata Z. Rettig T. Schulze W. (1985). The ultrastructure of sensory nerve endings in the human knee joint capsule. *Anat. Embryol.* 172 265–275. 10.1007/BF00318974 4061868

[B20] Iwanaga T. Fujita T. Takahashi Y. Nakajima T. (1982). Meissner’s and Pacinian corpuscles as studied by immunohistochemistry for S-100 protein, neuron-specific enolase and neurofilament protein. *Neurosci. Lett.* 31 117–121. 10.1016/0304-3940(82)90102-1 6813774

[B21] Johnson K. O. (2001). The roles and functions of cutaneous mechanoreceptors. *Curr. Opin. Neurobiol.* 11 455–461. 10.1016/s0959-4388(00)00234-8 11502392

[B22] Kawahara G. (1952). Studien über die Lamellenkörperchen im Pankreas der Katze. *Arch. Histol. Cytol.* 3 189–199. 10.1679/AOHC1950.3.189

[B23] Krause W. (1880). Die Nervenendigung innerhalb der terminalen Körperchen. *Archiv. Mikrosk. Anatomie* 19 53–136. 10.1007/BF02952690

[B24] Kumamoto K. Ohkawa Y. Ebara S. Matsuura T. Takeda H. (1988). Pacinian corpuscles in pancreas of the cat. *Meiji Coll. Oriental Med.* 4 43–50.

[B25] Landon D. N. Wiseman O. J. (2001). A Pacinian corpuscle in the human bladder lamina propria. *J. Neurocytol.* 30 457–464. 10.1023/a:1015624713894 12037462

[B26] Li R. Yang T. Zhang M. Ren K. Li J. Yi S. Q. (2024a). A new histopathological phenomenon: Pancreatic islet cell loss in the elderly population. *Dig. Liver Dis.* 56 1039–1045. 10.1016/j.dld.2023.11.031 38065700

[B27] Li R.-J. Yang T. Zeng Y.-H. Natsuyama Y. Ren K. Li J. (2024b). Impacts of different pancreatic resection ranges on endocrine function in *Suncus murinus.* *World J. Gastrointest. Surg* 16, 2308–2318. 10.4240/wjgs.v16.i7.2308 39087135 PMC11287669

[B28] Liu H. P. Leong S. K. Tay S. S. (1994). Localization of NADPH-diaphorase positive neurons in the pancreas of the mouse, rat, chick, kitten and monkey. *J. Hirnforsc.* 35 501–510. 7533810

[B29] Martín-Alguacil N. Aardsma N. Litvin Y. Mayoglou L. Dupré C. Pfaff D. W. (2011). Immunocytochemical characterization of pacinian-like corpuscles in the labia minora of prepubertal girls. *J. Pediatr. Adolesc. Gynecol.* 24 353–358. 10.1016/j.jpag.2011.06.005 21906975

[B30] Medlicott S. A. C. Larsen E. T. Gao Y. Trpko K. (2019). Pacinian corpuscle in the prostate: Fact – not fiction. *Hum. Pathol. Case Rep.* 15 71–72. 10.1016/j.ehpc.2018.12.001

[B31] Miclaus V. Catoi C. Oana L. Damian A. Ruxanda F. Rus V. (2011). *Observations Regarding the Presence and Distribution of Vater Pacini corpuscles from Pancreas in Cat.* Available online at: http://www.univagro-iasi.ro/simpozion_med/Revista/ (accessed March 12, 2025).

[B32] Morishita S. Sai K. Maeda S. Kuwahara-Otani S. Minato Y. Yagi H. (2018). Distribution of Pacini like lamellar corpuscles in the vascular sheath of the femoral artery. *Anat. Rec.* 301 1809–1814. 10.1002/ar.23934 30294881

[B33] Onishi Y. Hisato H. Maeda S. Minato Y. Kuwahara O. S. Yagi H. (2021). Relationship between lamellar sensory corpuscles distributed along the upper arm’s deep arteries and pulsating sensation of blood vessels. *J. Anat.* 239 101–110. 10.1111/joa.13398 33527396 PMC8197943

[B34] Pai S. A. (2017). Ectopic Pacinian corpuscle in the prostate. *Int. J. Surg. Pathol.* 25 609–610. 10.1177/1066896917705200 28420304

[B35] Paré M. Albrecht P. J. Noto C. J. Bodkin N. L. Pittenger G. L. Schreyer D. J. (2007). Differential hypertrophy and atrophy among all types of cutaneous innervation in the glabrous skin of the monkey hand during aging and naturally occurring type 2 diabetes. *J. Comp. Neurol.* 501 543–567. 10.1002/cne.21262 17278131

[B36] Polski J. M. Spreen A. N. (2005). Pacinian corpuscle in human lymph node. *Lymphology* 38 18–19. 15856682

[B37] Proshchina A. E. Krivova Y. S. Leonova O. G. Barabanov V. M. Saveliev S. V. (2018). *Development of Human Pancreatic Innervation.* London: InTech. 10.5772/intechopen.77089

[B38] Standop J. Ulrich A. Schneider M. B. Andrén-Sandberg A. Pour P. M. (2001). Pacinian corpuscle in the human pancreas. *Pancreas* 23 36–39. 10.1097/00006676-200107000-00005 11451145

[B39] Stecco C. Gagey O. Belloni A. Pozzuoli A. Porzionato A. Macchi V. (2007). Anatomy of the deep fascia of the upper limb. Second part: Study of innervation. *Morphologie* 91 38–43. 10.1016/j.morpho.2007.05.002 17574469

[B40] Stecco C. Porzionato A. Nacchi V. Tiengo C. Parenti A. Aldegheri R. (2006). Histological characteristics of the deep fascia of the upper limb. *Ital. J. Anat. Embryol.* 111 105–110. 10.1016/j.jbmt.2008.04.041 16981399

[B41] Sugai N. Cho K. H. Murakami G. Abe H. Uchiyama E. Kura H. (2021). Distribution of sole Pacinian corpuscles: A histological study using near-term human feet. *Surg. Radiol. Anat.* 43 1031–1039. 10.1007/s00276-021-02685-x 33471166

[B42] Uguen A. (2018). Another case of Pacinian corpuscle in a lymph node. *Anat. Rec.* 301 561–562. 10.1002/ar.23765 29281859

[B43] Varga I. Nosál M. Babál P. (2020). Ectopic lamellar Pacinian corpuscle within the thymus. Atypical or abnormal location? *Rom. J. Morphol. Embryol.* 61 273–276. 10.47162/RJME.61.1.33 32747922 PMC7728121

[B44] Zimmerman A. Bai L. Ginty D. D. (2014). The gentle touch receptors of mammalian skin. *Science* 346 950–954. 10.1126/science.1254229 25414303 PMC4450345

[B45] Zimny M. L. Wink C. S. (1991). Neuroreceptors in the tissues of the knee joint. *J. Electromyogr. Kinesiol.* 1 148–157. 10.1016/1050-6411(91)90031-Y 20870506

[B46] Ziolkowski L. H. Nikolaev Y. A. Chikamoto A. Oda M. Feketa V. V. Monedero-Alonso D. (2024). Structural and functional dissection of the Pacinian corpuscle reveals an active role of the inner core in touch detection. *bioRxiv [Preprint]* 10.1101/2024.08.24.609509 39253434 PMC11383032

