# Supplementary figures and images for: Comparative study on the distribution of Pacinian corpuscles in the pancreas
Source: Front Neuroanat. 2025 Aug 13;19:1593682. doi: 10.3389/fnana.2025.1593682 (PMC12380800; doi:10.3389/fnana.2025.1593682)

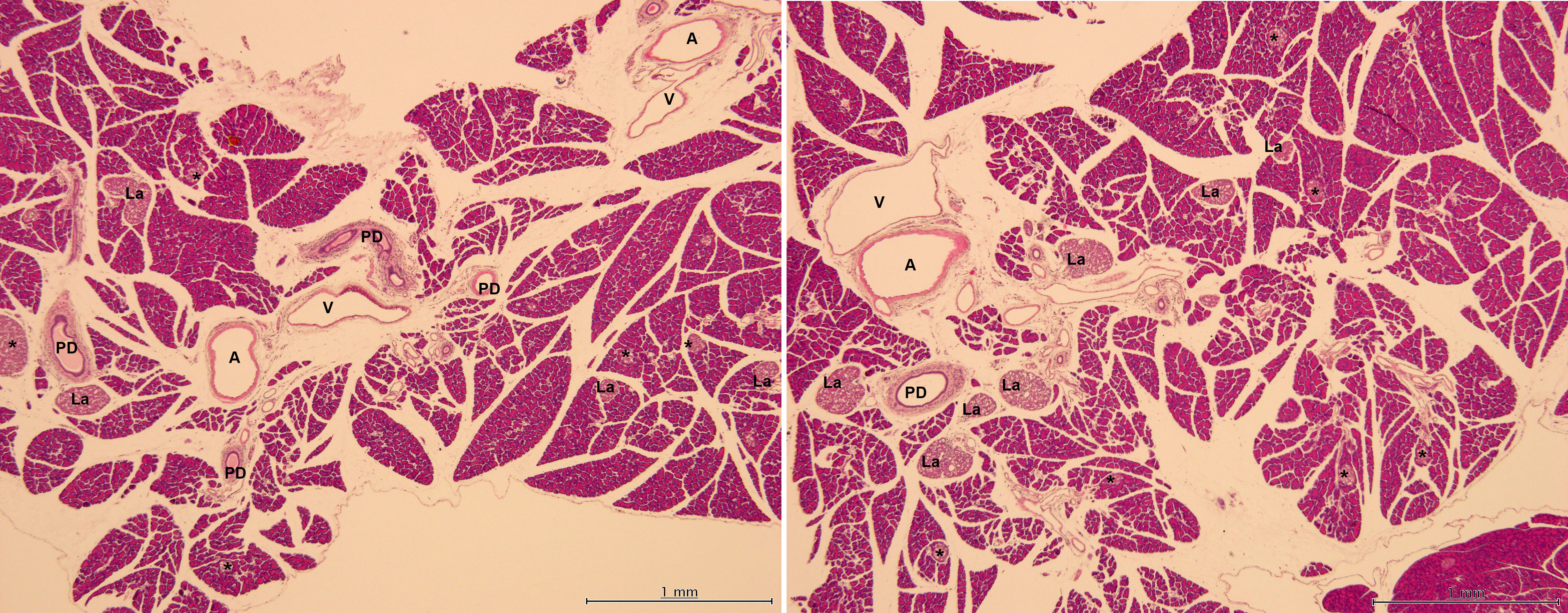

Supplement: Supplementary Figure 1 — HE staining of the pancreas of S. murinus. No PCs were observed in the pancreas of S. murinus. La, islets of Langerhans; *, small islets of Langerhans; PD, pancreatic duct; A, artery; V, vein. [file Image_1.jpeg]
